# 36. Clinical Features of and Risk Factors for 30-day Readmission after an Initial Hospitalization with COVID-19

**DOI:** 10.1093/ofid/ofab466.036

**Published:** 2021-12-04

**Authors:** Elisa Akagi Fukushima, Claudia Villatoro Santos, Mamta Sharma, Susan M Szpunar, Louis Saravolatz, Ashish Bhargava

**Affiliations:** 1 ST JOHN HOSPITAL, DETROIT, Michigan; 2 Ascension | St John Hospital & Medical Center, Grosse Pointe Woods, MI; 3 Ascension St. John Hospital, MI; 4 Ascension St John, Grosse Pointe Woods, MI

## Abstract

**Background:**

Little is known about risk factors for readmission after COVID-19 hospitalizations. Knowledge of these factors may help to identify patients at increased risk and may help to prevent these rehospitalizations.

**Methods:**

This historical cohort study was conducted at a tertiary care academic medical center. We included COVID-19 cases diagnosed by reverse-transcriptase polymerase-chain-reaction (RT-PCR) assay between March 8^th^ and June 14^th^, 2020. Patients readmitted within 30 days were identified. Using the electronic medical record, we collected data on demographic and clinical information. Data were analyzed using Student’s t-test, the chi-squared test and multivariable logistic regression.

**Results:**

We included 391 patients who survived after the index hospitalization for COVID-19. The readmission rate was 13.3% (52/391). The mean time to readmission was 9.2 ± 7.9 days. The mean age (±SD) was 66.3 ± 18.6 years, 44.2% were male, and 78.8% were black/African-American. The most common presenting complaint was shortness of breath (50%). The most frequent diagnosis during the readmission was infectious process (57.7%). The mortality rate on readmission was 11.5%. Patients with a 30-day readmission were older than those not readmitted, mean age (±SD) 66.3 ± 18.6 vs. 61.0 ± 16.0, respectively (p=0.03). Readmitted patients also had a higher prevalence of heart failure and renal disease as comorbidities. Elevated alanine aminotransferase (AST) and low albumin level were also associated with readmission (Table 1). Intensive care unit (ICU) admission or mechanical ventilation during the index admission did not increase the risk of readmission. From multivariable analysis, independent predictors of 30-day readmission were higher Charlson score (p=0.004), higher creatinine on admission in the index hospitalization (p=0.009), and presence of rhabdomyolysis during the index hospitalization (p=0.039) (Table 2).

Table 1. Univariable Analysis of Predictors for Readmission within 30 days from COVID-19 Infection

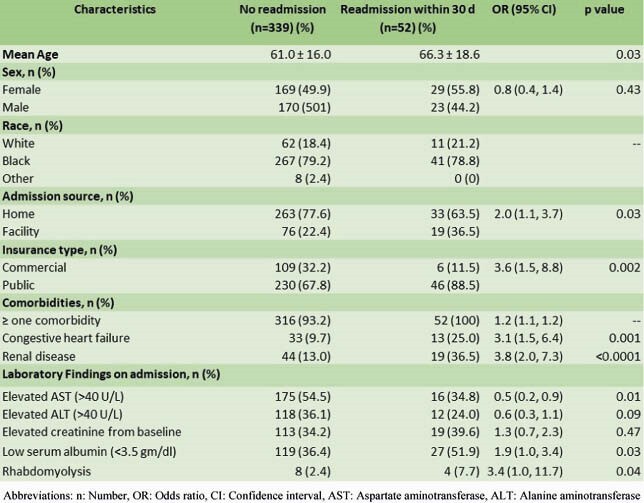

Table 2. Multivariable Analysis of Predictors for Readmission within 30 days from COVID-19 Infection

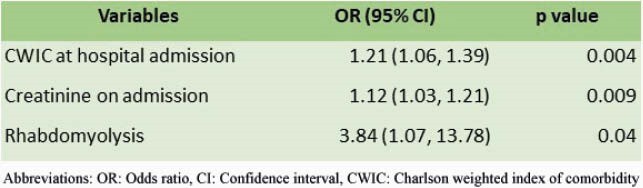

**Conclusion:**

In our cohort, infectious etiologies were common among those readmitted within 30 days of COVID-19. A higher Charlson score, acute renal failure, and rhabdomyolysis during the index admission were independent predictors of a 30-day readmission. Further studies are required to investigate these contributing factors.

**Disclosures:**

**All Authors**: No reported disclosures

